# Evaluation of the Transverse Crack Depth of Rail Bottoms Based on the Ultrasonic Guided Waves of Piezoelectric Sensor Arrays

**DOI:** 10.3390/s22187023

**Published:** 2022-09-16

**Authors:** Yuan Yang, Ping Wang, Tian-Lang Song, Yi Jiang, Wen-Tao Zhou, Wei-Lei Xu

**Affiliations:** 1 College of Automation Engineering, Nanjing University of Aeronautics and Astronautics, Nanjing 211106, China; 2 The Key Laboratory of Non-Destructive Testing and Monitoring Technology for High-Speed Transport Facilities of the Ministry of Industry and Information Technology, Nanjing 211106, China; 3 College of Electrical Engineering, Nanjing Vocational University of Technology, Nanjing 210023, China; 4 Nantong Sizhen Electronic Technology Co., Ltd, Nantong 226000, China

**Keywords:** transverse crack, rail bottom, high-frequency ultrasonic guided wave, piezoelectric sensor arrays, multi-value domain features, multi-path combined reconstruction, back propagation neural network

## Abstract

A method based on the high-frequency ultrasonic guided waves (UGWs) of a piezoelectric sensor array is proposed to monitor the depth of transverse cracks in rail bottoms. Selecting high-frequency UGWs with a center frequency of 350 kHz can enable the monitoring of cracks with a depth of 3.3 mm. The method of arranging piezoelectric sensor arrays on the upper surface and side of the rail bottom is simulated and analyzed, which allows the comprehensive monitoring of transverse cracks at different depths in the rail bottom. The multi-value domain features of the UGW signals are further extracted, and a back propagation neural network (BPNN) is used to establish the evaluation model of the transverse crack depth for the rail bottom. The optimal evaluation model of multi-path combination is reconstructed with the minimum value of the root mean square error (RMSE) as the evaluation standard. After testing and comparison, it was found that each metric of the reconstructed model is significantly better than each individual path; the RMSE is reduced to 0.3762; the coefficient of determination R^2^ reached 0.9932; the number of individual evaluation values with a relative error of less than 10% and 5% accounted for 100% and 87.50% of the total number of evaluations, respectively.

## 1. Introduction

**Figure 1:**
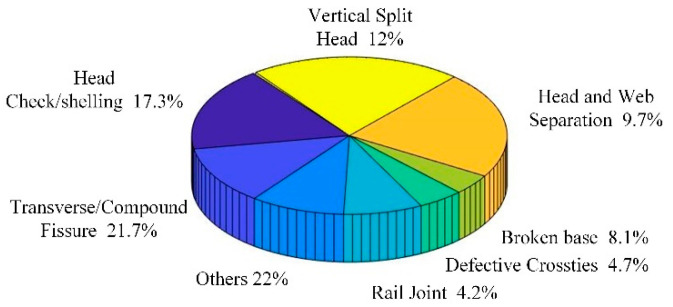
Accidents caused by different types of rail damage between 2005 and 2014.

**Figure 2:**
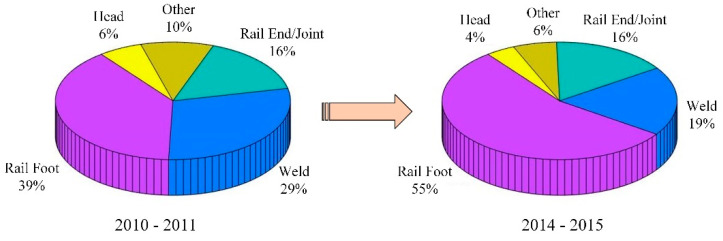
Distribution of the main locations of rail fractures in the UK railway network.

Railways are an important part of our infrastructure and play a key role in the overall transportation system. As rails carry trains, their safety plays a vital role in the safe operation of these vehicles. The actual operating environment of rails is complex, and the risk of rail fracture increases due to long-term high loads. Rail fracture is caused by many reasons, including rail bottom transverse cracks, bolt holes, weld failure, internal defects, and so on. According to the report published by the Office of the Federal Railroad Administration on railway accidents in the United States from 2005 to 2014, transverse cracks caused the biggest amount of railway accidents—21.7% of the 2653 accidents were due to transverse cracks [1,2,3]. The causes of accidents resulting from various types of rail damage and their proportion distribution are shown in Figure 1. A rail is structurally divided into three areas: rail head, rail web and rail bottom. Compared with the rail head and rail web, the rail bottom area is a detection blind area for existing rail damage detection-technology due to the particularities of the rail structure and installation. However, damage to the bottom of the rail is very harmful to the health of the rail structure, and it can easily cause the rail to break if it is not discovered and repaired in time. Figure 2 shows the causes and proportions of rail route breaks in the UK rail network for 2010–2011 and 2013–2014 [1,4,5]. From the above data, it can be seen that rail bottom defects are the main cause of rail fractures, attributed to 39% and 55% of all fractures that occurred in the two statistical intervals of 2010–2011 and 2013–2014, respectively. By comparing the data in Figure 1 and Figure 2, it can be concluded that transverse cracks on the rail bottom are one of the main causes of rail fractures, and it is vital to detect and maintain them in a timely manner.

At present, non-destructive testing (NDT) technology is usually used for detecting rail damage. Commonly used NDT technologies include visual inspection, magnetic induction, eddy current, photothermal, ultrasonic, etc., [6,7,8,9,10,11]. However, existing NDT methods mainly focus on the inspection of the rail head and rail web, and little research has been conducted on the detection of rail bottom defects, especially for the edge of the rail bottom that is far away from the rail web [12].

Although, in recent years, some scholars have carried out research on rail bottom damage. Jia [13] proposed detecting rail foot defects using infrared heat waves. According to the theory of heat waves, defects will affect the temperature distribution of rail surfaces, which will help achieve the purpose of detecting rail defects. Pathak et al., [14] proposed using the finite element simulation technology based on laser-induced ultrasonic-guided-wave (UGW) propagation to detect rail bottom defects by simulating different frequencies and different sensor positions. Hu et al., [15] proposed a method for the rapid detection of rail bottom cracks using B-scan imaging technology. This method generates shear-guided waves through electromagnetic ultrasonic transducers (EMAT) to perform B-scan imaging of transverse cracks in the rail bottom. These methods are used to test the bottom of rails through NDT, which are regularly inspected and maintained by workers to ensure their health. However, the NDT technologies need to occupy a long detection skylight, and the manual detection efficiency is low. Further, the rail fracture may occur during the non-detection period, having gone from a small defect to a complete rail fracture. Therefore, compared with the NDT method, the monitoring method is more suitable for the detection of damage to rail bottoms, especially to certain special parts such as the wing rail and the tip rail of the switch, without affecting the normal operation of the railway, where the damage can be identified in advance.

Monitoring methods can be divided into passive monitoring and active monitoring. Commonly used sensors for passive monitoring include accelerometers, strain gauges, and fibre–optic sensors. Accelerometers are often used to acquire structural global information such as modal data (frequency, mode shape, etc.). They are however insensitive to small damage which is a local phenomenon and therefore does not significantly modulate the global structural features. Strain gauges provide localised measurement, but they are good at capturing static or dynamic measurands at a relatively low variation rate only. Fibre–optic sensors measure local strain as well, but directivity and embeddability are factors that can influence the measurement accuracy to some extent [16]. UGW detection technology represents a commonly used active monitoring method. Compared with traditional ultrasonic waves, UGWs have a long propagation distance, fast propagation speed and high detection efficiency. They do not need to use train running time for the detection process and can enable all-weather online monitoring of rail–bottom cracks by being fixed on the rail for long periods of time. When UGWs encounter a crack, a reflected echo is generated, and the transmitted wave is attenuated, accompanied by a complex mode-conversion phenomenon in the process. Through the analysis and processing of the collected guided wave signals, we can judge whether the rail has cracks and other damage and can obtain specific damage information such as location and size [16]. At the beginning of the 21st century, Rose et al., [12] were the first to propose the application of UGWs for the detection of rail defects. In recent years, Xing et al., [17] proposed a defect localisation method based on the UGW single-mode algorithm to evaluate the defect location. Serafeim et al., [18] combined the UGW monitoring method with the Support Vector Machines classification method to realise the rapid detection of rail bottom damage. The excitation frequency signal of the UGWs was 100 kHz. Deng et al., [19] proposed a segmented principal component analysis method to extract the features of the signals collected by the sensors at different positions and used SVM to quickly classify the cracks, corrosion, transverse cracks and other defects on the rail head surface. Hu et al., [20] realised crack location monitoring of 14 mm at the bottom of the rail through active and passive UGWs. The aforementioned findings indicate that UGW technology has positive outcomes for rail crack monitoring. However, in the existing studies, the objects inspected using UGWs contain defects whose depths are in centimetres, and there has been little monitoring research on rail bottom transverse cracks. Therefore, it is necessary to monitor the transverse cracks at the bottom of the rail that are millimetres in depth to ensure the health of the rail and the safety of railway operations.

In summary, this paper adopts the method involving piezoelectric sensor arrays receiving and transmitting high-frequency UGWs combined with a back propagation neural network (BPNN) to evaluate the depth of the transverse cracks in rail bottoms. First, a new layout for the piezoelectric sensor arrays is proposed, where a 4 × 4 piezoelectric sensor array is placed on the upper surface and side of the rail bottom to ensure the comprehensive monitoring of transverse cracks at different depths in the rail bottom. High-frequency UGWs were selected to monitor millimetre-scale cracks and improve monitoring sensitivity. Further, the guided wave signals of each path were extracted and recombined with multi-feature extraction and combined with a BPNN. Next, the evaluation model was constructed based on multi-path reconstruction, and, finally, the high accuracy evaluation of transverse crack depth was realised.

The rest of the paper is arranged as follows: Section 2 describes the basic theories and methods employed in this paper, including the theory of UGWs, the selection of the excitation frequency, the arrangement and simulation of the piezoelectric sensor array, the principle of the BP neural network and the evaluation index of the evaluation model. Section 3 describes the experimental system and process in detail. Section 4 presents the definition and extraction of the multi-range features of multipath guided wave signals. Section 5 analyses the method involving the transverse–crack–depth–evaluation model based on a BPNN and multipath information reconstruction. Section 6 provides a summary of this paper. Finally, Section 7 provides an outlook for future work.

## 2. Materials and Methods

### 2.1. UGW Theory

**Figure 3:**
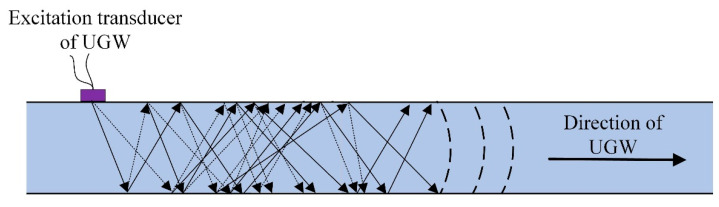
Schematic diagram of the propagation of UGWs in a plate structure.

The UGW is a special kind of acoustic wave generated by the continuous emission, refraction and conversion of longitudinal and transverse waves with the boundary of the medium when the acoustic wave propagates in the waveguide medium. An ultrasonic wave that propagates through a waveguide medium is called a UGW; examples include Lamb waves and Rayleigh waves. A UGW propagating through a plate is called a Lamb wave; it reflects back and forth between the boundaries of the plate and propagates forward, as shown in Figure 3. The rail is a complex structure, and the bottom area of the rail can be equated to a plate-like structure [21].

According to the different distribution patterns of the vibration displacement of the particles in the plate, Lamb waves can be divided into a symmetric mode and an antisymmetric mode. When the symmetrical Lamb wave propagates through the plate, the particle in the centre of the plate moves longitudinally, while the particles on the upper and lower surfaces both vibrate elliptically with opposite phases and symmetry than the centre; their dispersion equations are represented by Equations (1) and (2) [22,23].

(1) Symmetric mode: tan(qh)tanph=−4k2pqq2−k22

(2) $$\mathrm{Antisymmetric}\;\mathrm{mode}\text{: }\frac{\tan(\mathrm{qh})}{\tan\left(\mathrm{ph}\right)}=-\frac{{\left(q^{2}-k^{2}\right)}^{2}}{4k^{2}\mathrm{pq}}$$

(3) $$\mathrm{Among}\;\mathrm{them}\text{: }p^{2}=\frac{\omega^{2}}{C_{L}^{2}}-k^{2}$$

(4) $$q^{2}=\frac{\omega^{2}}{C_{T}^{2}}-k^{2}$$

(5) $$k=\frac{\omega }{C_{p}}=\frac{2\pi f}{C_{p}}$$

(6) $$h=\frac{d}{2}$$

In the above formulas, k is the wave number; $C_{P}$ is the phase velocity of the Lamb wave; $C_{L}$ is the longitudinal wave velocity; the longitudinal wave speed in the rail is 5950 m/s; $C_{T}$ is transverse wave velocity; the transverse wave speed in the rail is 3260 m/s; finally, ω is the circular frequency, and d is the thickness of the rail bottom. Solving Equations (1) and (2) can provide the relationship between the Lamb wave number k and the circular frequency ω and then reveal the relationship between frequency f and phase velocity $C_{P}$. Finally, the numerical solution of the dispersion curve can be obtained.

The group velocity and phase velocity of UGWs are the characteristic quantities that must be paid attention to when studying these waves. The group velocity refers to the propagation velocity of a group of waves with similar frequencies, while the phase velocity refers to the propagation velocity of a certain frequency harmonic [23]. The group velocity and UGW frequency determine the wavelength of the UGW, which, in turn, determines the damage identification accuracy of the UGW. The relationship between the group velocity and phase velocity is shown in Equation (7).

(7) $$C_{g}=\frac{d\left({\mathrm{kC}}_{p}\right)}{d_{k}}=C_{p}+k\frac{{\mathrm{dC}}_{p}}{\mathrm{dk}}$$

**Figure 4:**
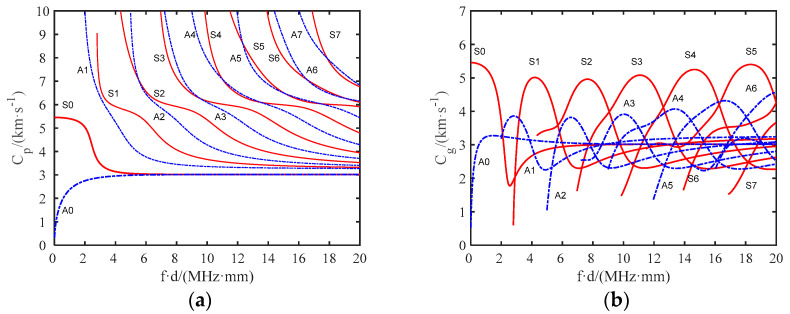
(**a**) Dispersion curve of the phase velocity; (**b**) dispersion curve of the group velocity.

During the propagation of UGWs through the rail bottom, geometric dispersion and multiple waves of different frequencies will appear, and the propagation speed changes with a change in frequency. This phenomenon is called guided wave dispersion. The dispersion curves of group velocity and phase velocity in the rail bottom area can be obtained through calculations using Equations (1)–(7) and are shown in Figure 4a,b.

### 2.2. Selection of the Excitation Frequency

**Figure 5:**
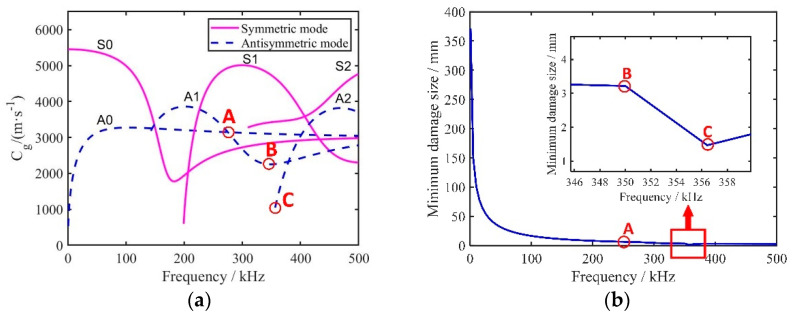
(**a**) Dispersion curve of rail bottom; (**b**) excitation frequency and minimum size of detectable damage.

In NDT and structural health monitoring, the accuracy of damage identification is determined by the wavelength of the UGWs. In the damage identification accuracy of UGW, the damage size l must be greater than half of its wavelength λ [16], as shown in Equation (8). According to this benchmark principle, the half section of the rail bottom is approximately trapezoid in shape, and its middle thickness is about 14 mm. Figure 5a shows the group velocity dispersion curve of the UGW in the rail bottom in the frequency range of 0–500 kHz. The antisymmetric mode of the UGW in the frequency range of 0–500 k was extracted. The relationship between the excitation frequency and the detectable damage size was obtained by dividing the maximum wave velocity of each frequency point by the corresponding frequency value, as shown in Figure 5b. The excitation frequency is inversely proportional to the detectable damage size. To optimise the selection of the excitation frequency, three special points, A, B and C, were selected for auxiliary frequency selection. The frequency at point A was relatively low. Although the number of modes was small, its damage identification accuracy was low, and the wave velocity difference between the modes was also small, which is not conducive to the identification of small defects. Point B was the point with the largest wave velocity difference between modes, and this frequency point was conducive to mode extraction. The new antisymmetric mode at point C was about to appear. Although the recognition accuracy was high, the number of modes was large at this point in time, which made mode selection more difficult. After comprehensive consideration, based on its larger wave velocity difference, smaller number of modes and smaller detectable damage size, point B (350 kHz) was selected as the excitation frequency as a compromise. Theoretically, at this frequency, the crack identification accuracy of the UGW is up to 3.3 mm.

(8) $$l\geq \frac{1}{2}\lambda =\frac{c}{2f}$$

### 2.3. Design of Piezoelectric Sensor Array

**Figure 6:**
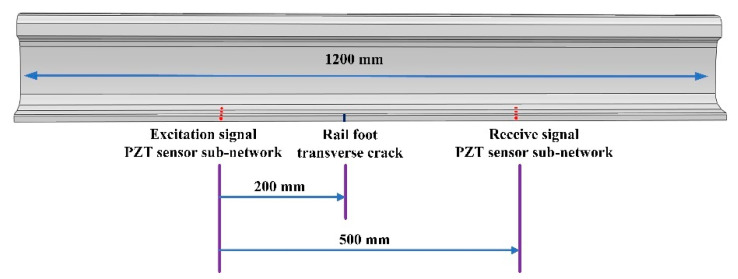
Schematic diagram of piezoelectric sensor array on rail bottom.

The arrangement of the piezoelectric sensor array is shown in Figure 6. The piezoelectric sensor array provides a simple and efficient method to monitor the bottom area of the rail. The array presented in this paper used a piezoelectric ceramic sheet (PZT) as the ultrasonic transducer. The specific model is PZT–5A, which has the advantages of high sensitivity and good stability [24]. It was composed of two sub-arrays, one of which was used for the excitation signal of the UGW and the other for the receiving signal of the UGW. Each sub-array was composed of four PZT–5As, where three were placed on the upper surface of the rail bottom, and one was placed on the bottom-side edge of the rail. Further, the interval between the two sub-network arrays was 500 mm. There were three PZT–5As on the upper surface of the rail bottom, which could effectively monitor various types of damage to the rail bottom. The damage mainly considered in this paper is the transverse cracks in rail bottoms.

**Figure 7:**
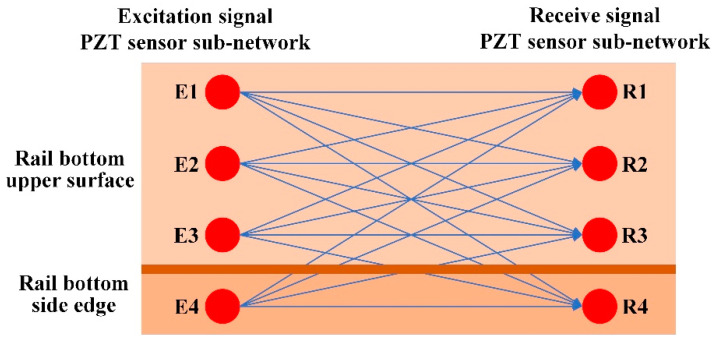
Schematic diagram of piezoelectric sensor arrays multipath monitoring.

Compared with the piezoelectric sensor array on the upper surface of the rail, the PZT–5A on the bottom edge was more sensitive to the transverse micro-cracks that occur in the rail bottom, which could effectively improve the identification accuracy. The 4 × 4 piezoelectric sensor array could form 16 paths, and these paths could monitor the transverse cracks in the bottom of the rail. The path diagram is shown in Figure 7.

**Figure 8:**
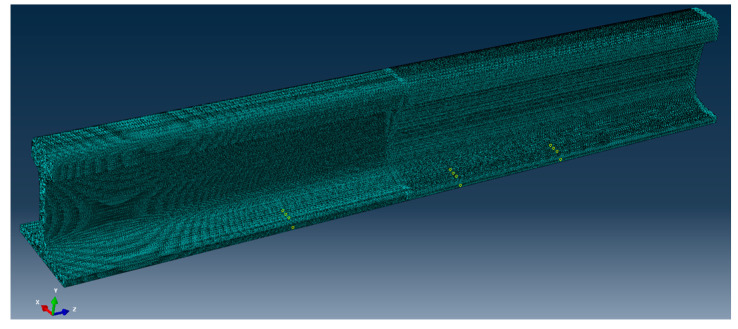
Grid diagram of rail model.

**Figure 9:**
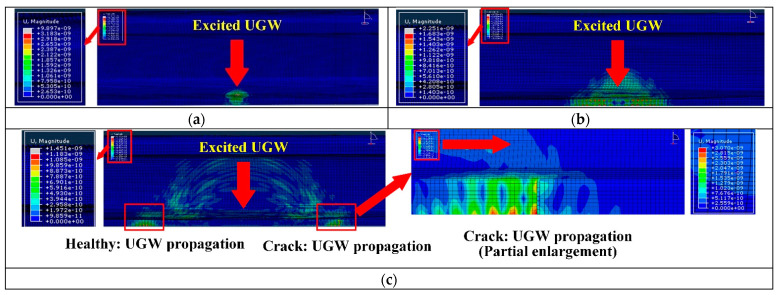
Propagation cloud diagram of UGW on rail bottom at different times; (**a**–**c**) are the propagation nephograms of ultrasonic guided waves at 15 μs, 39 μs, and 81 μs, respectively.

**Figure 10:**
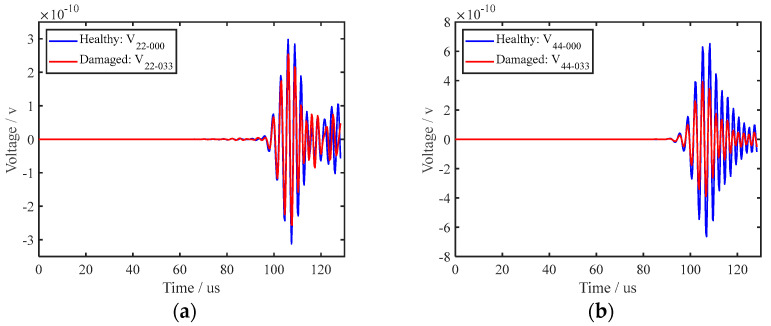
Signal comparison of UGW with and without transverse cracks in the rail bottom; (**a**,**b**) are paths E2-R2 and E4-R4, respectively.

To verify the effectiveness of the piezoelectric sensor array arrangement, in this research, ABAQUS finite element analysis software is selected to model and simulate the piezoelectric sensor array. Compared with other finite element analysis software, this analysis software has a higher degree of professionalism in the field of structural health acoustic monitoring, and its working efficiency is high, and the computer resources occupied are relatively small. Many researchers choose ABAQUS to conduct relevant research when studying ultrasonic guided waves [25,26,27]. The ABAQUS 6.13 finite element analysis software was used for simulation, and the specific setting parameters of the simulation are shown in Table 1. A 1200 mm-long rail model was established, and a piezoelectric sensor array was placed at the bottom of the rail, which consisted of two sub-arrays: one sub-array was used for excitation, while the other was used for reception; the two piezoelectric sensor sub-arrays were placed 500 mm apart. The transverse crack (with a width of 1 mm, length of 12 mm and depth of 3.3 mm) of the rail was set 200 mm away from the excitation piezoelectric sensor sub-array to help illustrate the interaction between the UGW and the transverse crack damage. In the established model, the depth for the transverse crack was continuously increased. The experiment simulated depths of 3.3 mm, 5.3 mm, 7.5 mm, 9.0 mm, 11.0 mm, 13.0 mm, 15.0 mm and 17.5 mm for the transverse crack. Figure 8 is a grid diagram of the rail model, and Figure 9 shows the propagation cloud diagram of the UGW at different times for excitation point 2 in the piezoelectric sensing array on the upper surface of the rail bottom. Figure 9a is the propagation cloud diagram of the UGW at the time of 15 μs, while Figure 9b is the propagation cloud diagram of the UGW at the time of 39 μs. At this time, the UGW propagated from the excitation point along the rail bottom to both sides. Figure 9c is the propagation cloud diagram of the UGW at the time of 81 μs. It can be noted from observing the UGW encountering a transverse crack in the rail bottom that the propagation of the UGW is different for healthy and cracked rails. The simulation results show that when the UGW encounters the transverse crack in the rail bottom, it interacts with the crack, resulting in reflection, diffraction and other phenomena, and the energy of the direct wave is weakened. The time-domain signal collected by the piezoelectric sensor array is analysed again, and the received time-domain signal of the UGW is represented by $V_{\mathrm{mn}-\mathrm{xxx}}$ (mn refers to the corresponding position of the excited and received probe, and xxx is the marked crack size. For example, path E1–R1 with a crack depth of 3.3 mm can be denoted as 11-033). Taking the E2–R2 and E4–R4 paths as examples, the received signals of the UGWs in the non-destructive state of the rail are denoted as V_22-000_ and V_44-000_. When the crack depth at the bottom of the rail is 3.3 mm, the signals of ultrasonic guided waves are V_22-033_ and V_44-033_. Figure 10a,b show the signal comparison diagram of the UGW with and without cracks for the paths E2–R2 and E4–R4, with a focus on the first direct wave, namely the first wave peak. The results show that regardless of whether the piezoelectric sensor arrays are arranged on the upper surface of the rail bottom or the side edge of the rail bottom, they can effectively monitor the transverse cracks in the rail bottom.

**Figure 11:**
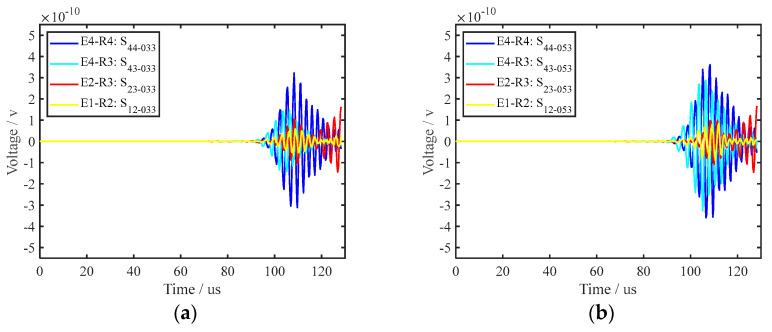
Signal amplitude variation in each path for cracks with small depths; (**a**,**b**) are the crack depths 3.3 mm and 5.3 mm, respectively.

**Figure 12:**
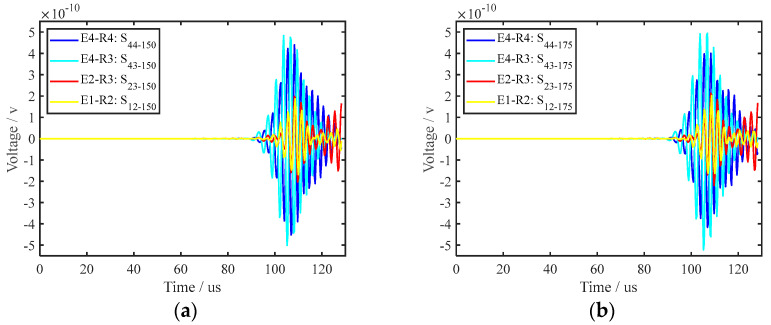
Signal amplitude variation in each path for cracks with large depths; (**a**,**b**) are the crack depths 15 mm and 17.5 mm, respectively.

**Figure 13:**
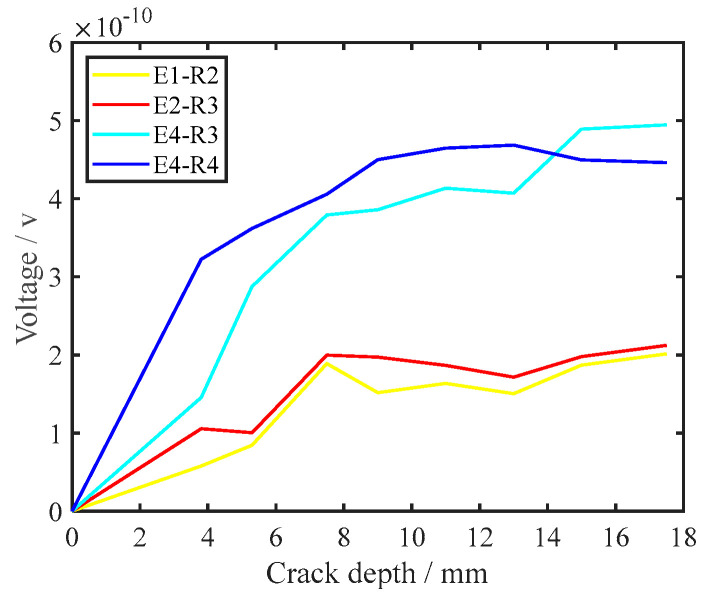
Signal amplitude variation at different crack depths in the same path.

By comparing the signal for a healthy rail with that for a crack in the bottom of the rail for the same path, the interaction between the crack and the UGW can be visually highlighted, which is beneficial for signal analysis. Therefore, differential processing was performed for all received UGW signals, and the formula is shown in Equation (9). By comparing the 16 path signals of transverse cracks with different depths, it was found that when the depth of the cracks is small, the PZT–5A on the side of the rail bottom could better characterise the damage state than the one on the upper surface of the rail bottom. When the transverse crack size is 3.3 mm and 5.3 mm, as shown in Figure 11a, b, the signal difference of the E1–R2, E2–R3, E4–R3 and E4–R4 paths is selected. As can be seen from Figure 11, when the depth of the transverse crack in the bottom of the rail was small, the PZT–5A arranged at the side of the rail bottom was more sensitive to the transverse crack. However, as the depth of the transverse crack increased, the PZT–5A on the side of the rail bottom provided a weaker representation of the crack depth information. The PZT–5A array placed on the upper surface of the rail bottom, the path composed of the PZT–5A on the upper surface and the PZT–5A on the side enhanced the characterisation of the crack depth information. Figure 12 shows the sensitivity of each path to transverse cracks when the cracks were large. Figure 12a,b show transverse cracks of 15.0 mm and 17.5 mm, respectively, and the E1–R2, E2–R3, E4–R3, and E4–R4 paths are also selected. It can be seen from Figure 13 that the path E4–R3 was the most sensitive to cracks when the transverse crack depth was larger. Since the UGW excited by the PZT–5A on the side first propagated along the side of the rail bottom, the UGW excited by the PZT–5A array on the upper surface of the rail propagated along the upper surface of the rail bottom. Figure 13 shows the attenuation of the UGW signal with different sizes of cracks when the wave encounters a crack, which also reflects the sensitivity of each path to the crack. It can be seen from the figure that when the transverse crack in the rail bottom was small, the PZT–5A sensitivity of the side was higher. However, as the depth of the transverse crack increased, due to the existence of the transverse crack in the rail bottom, the transmitted wave bypassing the crack became increasingly less and, finally, presented a constant value. The PZT–5A on the side reached a relatively constant value relative to that on the upper surface. In the monitoring area of the piezoelectric sensor array on the upper surface of the rail bottom, with an increase in crack depth, the UGW propagation was gradually affected, and its monitoring sensitivity also gradually increased. This further illustrates the effectiveness of the piezoelectric sensing array arrangement in this paper, which can comprehensively monitor and evaluate changes in transverse crack depth.

(9) $$S_{\mathrm{mn}-\mathrm{xxx}}=V_{\mathrm{mn}-\mathrm{xxx}}-V_{\mathrm{mn}-000}$$

Here, mn refers to the corresponding position of the excited and received probe, and xxx is the marked crack size. For example, path E1–R1 with a crack depth of 3.3 mm and 17.5 mm can be denoted as 11–033 and 11–175, respectively.

### 2.4. Principle of a BP Neural Network

A BPNN is a multi-layer feedforward network trained according to error backpropagation, which is one of the most widely used neural network models. The basic idea is that the gradient descent method and the gradient search technology are used to minimise the error mean square deviation between the actual output value and the expected value of the network [28].

**Figure 14:**
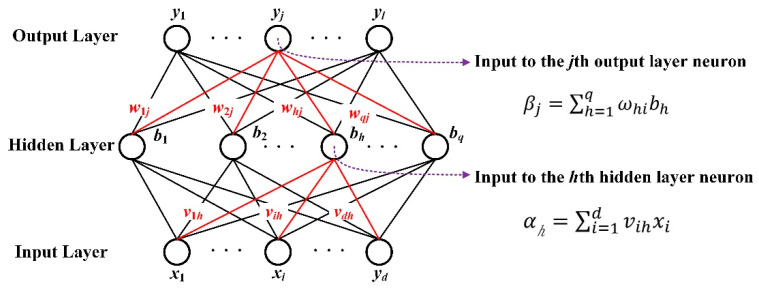
The model of BPNN.

The topology structure of the BPNN model includes an input layer, a hidden layer and an output layer. The input layer is mainly responsible for receiving external data. The hidden layer is the processing end of information, and the number of hidden layers can be set. Finally, the output layer refers to the output side of the information. The BPNN model is shown in Figure 14.

The learning process of the BPNN consists of two activities: forward propagation of the signal and backpropagation of the error. During forward propagation, the features of the sample are input from the input layer; the signal is then processed by each hidden layer and, finally, output by the output layer. Among these, the equation from the input layer to the hidden layer is Equation (10), and the equation from the hidden layer to the output layer is Equation (11).

From input layer to hidden layer:

(10) $$\alpha_{h}=\overset{d}{\underset{i=1}{\sum }}v_{\mathrm{ih}}x_{i}+\theta_{h}$$

From hidden layer to output layer:

(11) $$\alpha_{h}=\overset{d}{\underset{i=1}{\sum }}v_{\mathrm{ih}}x_{i}+\theta_{h}$$

At this time, the error between the actual output and the expected output of the network is calculated, and the error is shown using Equation (12). The error is transmitted back layer by layer from the last layer to obtain the error learning signal between each layer, and the error learning signal is used to correct the weight of the neurons in each layer. With the forward propagation of the signal and the reverse propagation of the error, the process of adjusting the weights of each layer is continuously carried out, and the weights of each layer are also constantly being adjusted. The weight update equation is represented by Equations (13) and (14). In the former, l is called the learning rate, and the update pace can be adjusted. An appropriate learning rate can make the target converge to the local minimum at an appropriate time. This process also serves as the process of network learning until the error decreases below a pre-set threshold or exceeds a pre-set maximum number of training iterations [29].

(12) $$\alpha_{h}=\overset{d}{\underset{i=1}{\sum }}v_{\mathrm{ih}}x_{i}+\theta_{h}$$

(13) $$\Delta \omega_{\mathrm{ij}}=\left(l\right){\mathrm{Ey}}_{k}$$

(14) $$\omega_{\mathrm{ij}}=\Delta \omega_{\mathrm{ij}}+\omega_{\mathrm{ij}}$$

### 2.5. Evaluation Metrics for Model Performance

To verify the credibility of the evaluation results, several measure metrics are proposed to evaluate the evaluation model performance, namely the root mean square error (RMSE), the coefficient of determination R^2^ and the proportion of the number of individual evaluation values whose relative errors are less than 10% and 5% in accounting for the total number of evaluations (denoted as P__Rr010_ and P__Rr010_). Here, the smaller the RMSE, the more accurate the model evaluation results. The coefficient of determination reflects the accuracy of the model fitting data. Generally, the coefficient of determination R^2^ ranges from 0 to 1. The closer the value is to 1, the higher the degree of interpretation of the independent variable to the dependent variable and the better the evaluation model fits the data. At the same time, the proportion of the number of individual evaluation values whose relative error is less than 10% and 5% account for the total number of evaluations. These two metrics further illustrate the accuracy of the evaluation of a single review. The expressions of each evaluation metric are shown in Table 2.

## 3. Experiments and Systems

### 3.1. The Experimental System

**Figure 15:**
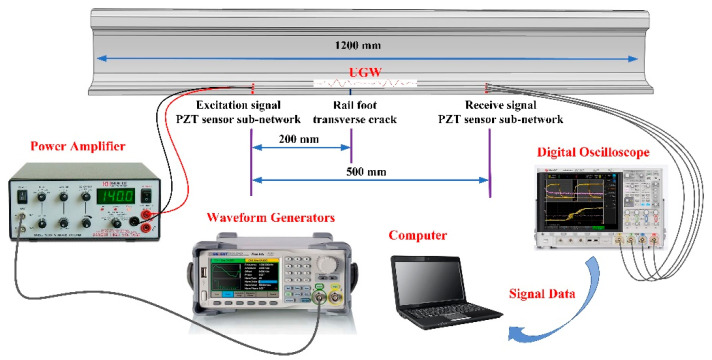
Schematic diagram of the experimental system.

**Figure 16:**
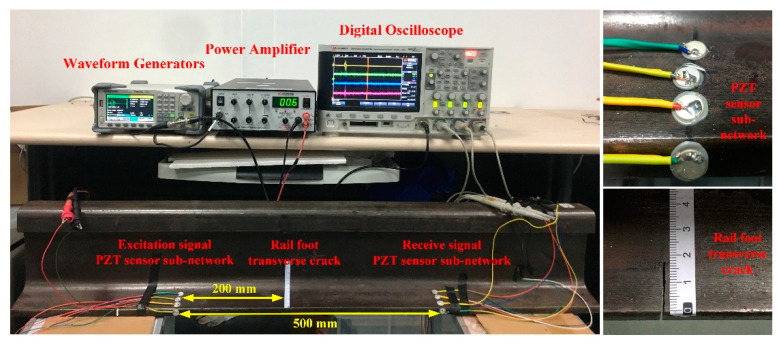
Diagram of the actual experimental system.

An experimental system was built to verify the effectiveness of the piezoelectric sensing array placed on the rail bottom area to evaluate the depth of transverse cracks. This experiment was carried out on a section of a real rail of 1200 mm length. It was a CHN60 rail, which is the most widely used type for high-speed rail lines. The centre frequency of the selected specification for the PZT–5A was 350 kHz, and the size of the PZT–5A was ∅10 mm∗0.75 mm. The arrangement of the piezoelectric sensor array is shown in Figure 15. The signal excitation of the UGW was composed of four PZT–5As named E1, E2, E3, and E4, which were excited in succession during operation. The signal reception of the UGW was carried out using four PZT–5As named R1, R2, R3, and R4, and this occurred simultaneously during operation. The distance between the excitation position and the receiving position of the UGW was 500 mm, and the transverse crack damage position of the rail bottom was 200 mm from the excitation position. The transverse cracks in the rail bottom were manually created using a hacksaw. The damage sizes were 3.3 mm, 5.3 mm, 7.5 mm, 9.0 mm, 11.0 mm, 13.0 mm, 15.0 mm and 17.5 mm. The whole system was composed of a waveform generator, power amplifier, digital oscilloscope and PZT–5A for ultrasound transducers. Among these, the signal generator was used to generate the required waveform of the excitation signal, the power amplifier was used to amplify the excitation signal, and the digital oscilloscope was used to collect and save the data, which was processed by the computer. The schematic diagram of the experimental system is shown in Figure 15, and the real experimental system is shown in Figure 16.

### 3.2. Experiment

**Figure 17:**
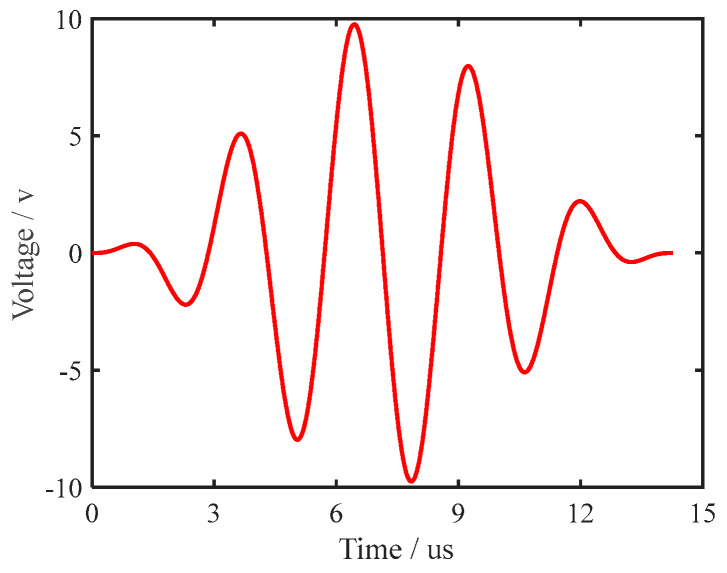
Excitation signal waveform with centre frequency of 350 kHz.

**Figure 18:**
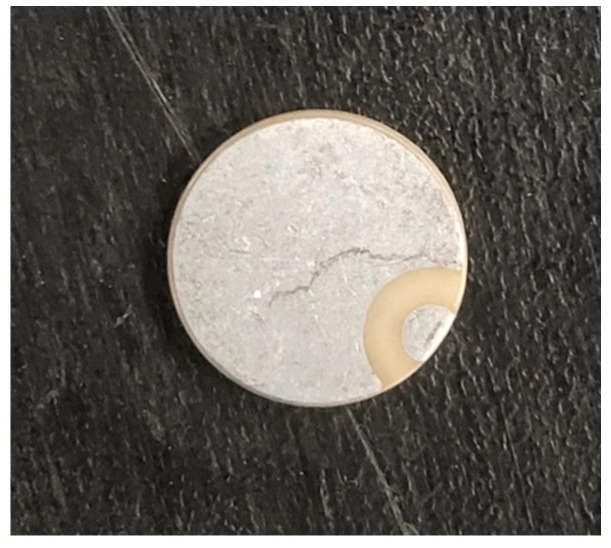
Picture of PZT–5A with the centre frequency of 350 kHz.

To reduce the dispersion of the UGW in the process of propagation, a narrow bandwidth signal is often selected as the excitation waveform. In this study, the signal generated by the sine wave modulated by the Hanning window was selected. The spectrum of the sine wave modulated by the Hanning window is narrow, which can effectively suppress the dispersion phenomenon of the UGW. A sine wave modulated by a five-period Hanning window was the most suitable excitation waveform for this experiment [30]. The waveform of the excitation signal is shown in Figure 17, and the centre frequency of the excitation signal was 350 kHz. The physical picture of PZT–5A is shown in Figure 18. The size of the PZT–5A was ∅10 mm∗0.75 mm. PZT–5A is equivalent to capacitive load, its driving voltage is 0 to 150 V.

After the system was built, the excitation signal with a frequency of 350 kHz was generated by the waveform generator. The initial peak value of the signal was 10 V, and the voltage rose to 144 V after being amplified by the power amplifier. For the healthy state of the rail, the PZT–5A numbered E1 was excited first; the PZT–5As numbered R1, R2, R3 and R4 received the UGW signal simultaneously. Next, the PZT–5As numbered E2, E3 and E4 were excited sequentially, and the PZT–5As numbered R1, R2 and R3 received the UGW signal at the same time as R4. In this way, the data of the non-destructive state of the rail under 16 paths of Em–Rn (m = 1, 2, 3 and 4, and n = 1, 2, 3 and 4) were obtained, and the data was saved 20 times under each path. After that, the rail bottom was manually sawed by a hacksaw to make transverse cracks. The data collection was carried out under the state of transverse cracks of different depths according to the same method and steps employed for monitoring the data collection of the rail in the healthy state, and the data was collected 20 times for each depth crack and each path.

In the whole experiment, with 16 paths, one healthy rail and eight kinds of rail bottom transverse crack damage states, the total volume of data comprised 2880 groups, where one path and eight kinds of damage data amount to 160 groups. Here, 60% of the data was used for model training, 20% was used as Test Set I for model optimisation, and the remaining 20% was used as Test Set II to test the optimised multi-path combination model.

## 4. Feature Definition and Extraction

**Figure 19:**
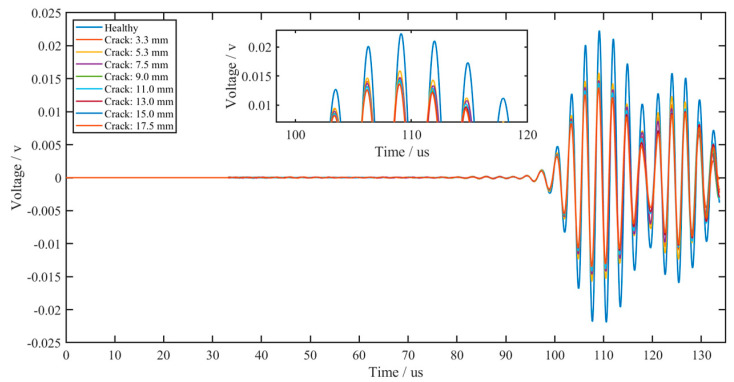
Signal waveforms of UGW collected at different crack depths (path E2–R2).

Figure 19 shows the signal waveforms of the UGW collected at different crack depths in the time range of 140 μs for the path E2–R2 in the experiment. The transverse crack depths of the rail bottom were 3.3 mm, 5.3 mm, 7.5 mm, 9.0 mm, 11.0 mm, 13.0 mm, 15.0 mm and 17.5 mm. It can be seen from Figure 18 that the monitoring signal waveforms caused by cracks at different depths of the rail bottom are roughly similar, and the signal amplitude only has a large attenuation when the crack first appears; then, with an increase in the crack depth, the signal amplitude changes very little. According to the elastic wave theory, due to the existence of a transverse crack defect on the rail bottom, the UGW will not only interact with the transverse crack on the rail but also reflect with the edge of the rail bottom. The corresponding wave packet will cause the amplitude change and the overlap of the final time domain signal. Therefore, the crack signal may be submerged, and it will become difficult to distinguish the depth of the transverse crack at the rail bottom using only the time domain waveform signal of the crack detection signal; thus, more characteristic information needs to be extracted.

It is well known that combining feature extraction with the time domain, frequency domain and time–frequency domain can significantly improve the detection capability of applied techniques in the field of NDT and monitoring. In this paper, feature extraction was carried out using the following three feature sets: time domain feature set $F_{time}^{*}$, frequency domain feature set $F_{frequency}^{*}$ and time–frequency domain feature set $F_{time–frequency}^{*}$. Therefore, the general feature set can be expressed as $F^{*}=\left\{F_{time}^{*},F_{frequency}^{*},F_{time-frequency}^{*}\right\}$.

The time domain features mainly include dimensional and dimensionless feature parameters. The main features include maximum value, minimum value, average value, square root amplitude, biased variance, standard deviation, root mean square, kurtosis, skewness, waveform factor, peak factor, impulse factor, margin factor and clearance factor. These time domain feature parameters and their expressions are shown in Table 3.

The frequency domain features can analyse signal problems more accurately. The commonly used frequency features in research mainly include the centroid frequency, mean frequency, root mean square frequency and root variance frequency. The frequency domain characteristic parameter expressions are shown in Table 4.

The time–frequency domain features can describe the frequency content of a signal over a period of time. For a given discrete time signal, the time–frequency analysis method can provide the frequency information corresponding to a certain time, which is a complex function of time and frequency. In recent years, researchers have found that wavelet analysis is particularly useful for extracting information from NDT and monitoring signals [31,32]. The signals of UGWs contain features of different resolutions. The wavelet transform uses wide and narrow windows to separate the slow and fast frequencies in the signals, such that the best time–frequency resolution is generated in all frequency ranges [18,33]. The wavelet transform decomposes the signal according to different resolutions, such that the original signal is decomposed into multiple sub-frequency bands, and can obtain wavelet singular entropy and wavelet energy entropy, along with other time–frequency domain features.

## 5. Evaluation Model Based on Multi-Path Reconstruction

**Figure 20:**
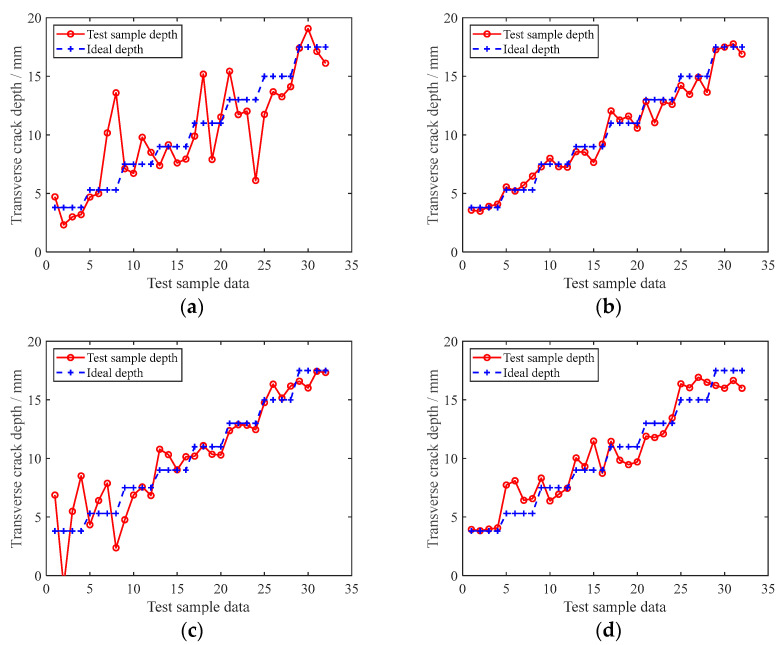
Test Set I test results for each path model; (**a**–**d**) are the paths of E1–R2, E2–R4, E3–R1, and E4–R4, respectively.

According to the feature extraction method described in Section 4, 60% of the data collected in the experiment was used as the training set, and the BPNN model in Figure 14 was used to establish the evaluation model of the transverse crack depth in the rail bottom. Given the design of the piezoelectric sensor array, there are 16 monitoring paths. The BPNN was used to establish the evaluation model of the rail bottom transverse crack depth for the corresponding paths, and Test Set I, which comprised 20% of the data volume, was used to test the evaluation model of each path. Figure 19 presents the estimated and ideal depths of transverse cracks in the test specimens for paths E1–R2, E2–R4, E3–R1 and E4–R4. Under the premise that the parameter settings of the BPNN and the method and environment of the collected data samples are the same, it can be seen from Figure 20 that the final evaluation results obtained for each path are quite different. Some paths have better evaluation results for small transverse crack depths, while others have better evaluation results for large transverse crack depths. The reason is as previously stated. When the rail transverse crack depth is different, the sensitivity of each path to the transverse crack is different. Therefore, it is proposed that the evaluation model of each small segment be optimised and screened using the piezoelectric sensor array and the multi-path combination be realised to more accurately evaluate the transverse crack depth of the rail bottom.

Using 60% of the data to train the generated model, the above Test Set I was used to test and evaluate the model, and the evaluation results provided a basis for the subsequent screening and reconstruction of the same using the piezoelectric sensor array. The process of path screening and reconstruction is shown in Figure 20. The evaluation results of the above paths are divided into eight segments, numbered from 1 to 8, and 16 paths can be denoted as Em–Rn (m = 1,2,3, and 4, and n = 1,2,3, and 4). As can be seen above, when the crack depth is different, the sensitivity of different paths to the crack is different, which affects the accuracy of the evaluation results. In this paper, by comparing the evaluation results of different paths, the corresponding path of the small segment with the best evaluation result for the same crack depth can be screened out. Finally, the path reconstruction of the transverse crack depth evaluation is carried out to form a multi-path combined evaluation model.

**Figure 21:**
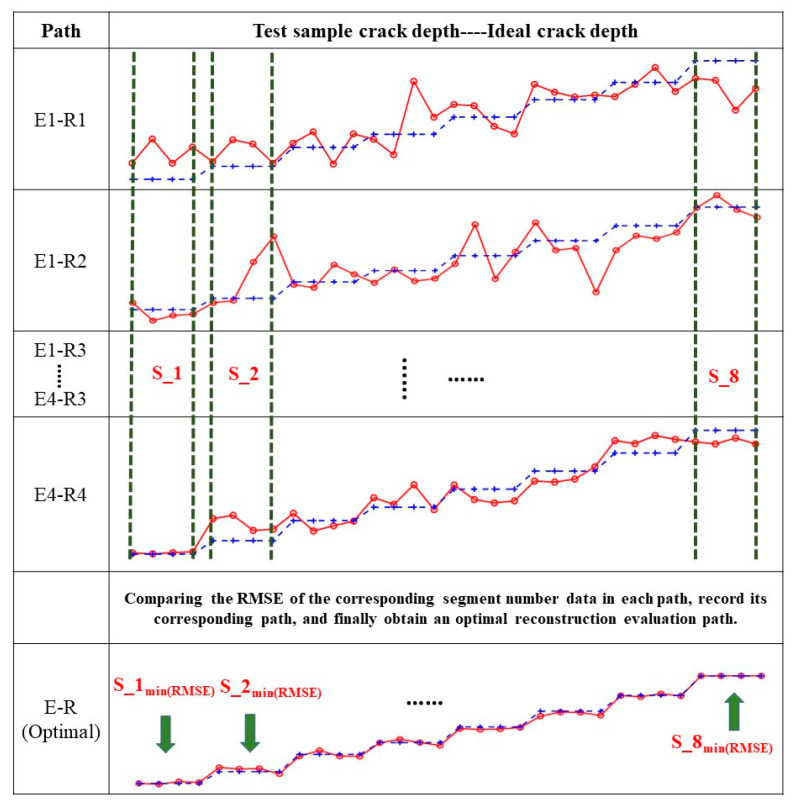
Guided map for the multi-path combined reconstruction model.

The specific method is as follows. The RMSE of each small segment evaluation result was calculated, and the RMSE of each corresponding small segment in the 16 paths was compared. The minimum value of the RMSE was used as the evaluation standard, the optimal segments were identified, and their corresponding path numbers were recorded. After comparison, eight optimal small–segment evaluation paths were obtained, which were reconstructed, and, finally, a multi–path combined rail–bottom transverse–crack evaluation model was obtained. The guided map for the multi-path combined reconstruction model is shown in Figure 21.

The remaining 20% of the data was taken as Test Set II, which was input into the 16 path models before reconstruction and the final model of multi-path combination after reconstruction for evaluation. The metrics of RMSE, the coefficient of determination R^2^ of the transverse crack evaluation data and the proportion of the number of single evaluation values whose relative error are less than 10% and 5% accounting for the total number of evaluations are shown in Table 5 and Table 6. The results show that the evaluation results of the reconstructed multi-path combined model are significantly better than the evaluation results of each individual path. The RMSE is reduced to 0.3762, and the coefficient of determination R^2^ is 0.9932. The number of individual evaluation values with a relative error of less than 10% and 5% accounted for 100% and 87.50% of the total number of evaluations, respectively.

## 6. Conclusions

In this paper, a method for monitoring and evaluating the depth of transverse cracks in rail bottoms based on a high-frequency UGW of piezoelectric sensing arrays is proposed. First, the optimal excitation frequency of 350 kHz was selected based on the dispersion characteristics of the UGW in the rail bottom, which can achieve a monitoring sensitivity of 3.3 mm depth cracks. A new arrangement of a 4 × 4 piezoelectric sensor array was adopted, and the piezoelectric sensors were arranged on the upper surface and the side of the rail bottom to form an array monitoring setup with 16 paths. Through simulation analysis, the array monitoring method proposed in this paper can realise the comprehensive monitoring of transverse cracks at different depths in a rail bottom compared with the piezoelectric sensor monitoring method that involves one receiving end and one sending end. To effectively extract information about the interaction between UGWs and cracks, this study performed multi-value domain feature extraction, including the time domain, frequency domain and time–frequency domain features, with a total of 54 features, and recombined all the features as the features of each individual path. The experimental samples for each path are randomly divided into the training set, Test Set I and Test Set II, according to the ratio 3:1:1. The evaluation results for each path were obtained by inputting the training set into a BPNN to establish the primary transverse crack evaluation model and then inputting the Test Set I into the evaluation model. The evaluation results for each path were divided into eight small segments, and the minimum value of the RMSE was used as the evaluation standard to select the optimal small segments and reconstruct the optimal transverse crack depth evaluation model for multi-path combination. Next, Test Set II was input into the reconstruction model for verification, and, finally, the accurate evaluation of transverse cracks at different depths in the rail bottom was realised. The evaluation results for each metric were significantly better than those for each individual path. The RMSE is reduced to 0.3762, and the coefficient of determination R^2^ is 0.9932, The number of individual evaluation values with a relative error of less than 10% and 5% accounted for 100% and 87.50% of the total number of evaluations, respectively.

## 7. Future Work

In this study, a piezoelectric sensor array was proposed to monitor transverse cracks in a rail bottom, and the feasibility and effectiveness of the technique were thoroughly verified. However, there are still some works to be further studied. First of all, the modules such as the excitation source and the power amplifier in the current system use the existing finished instruments, and the cost is relatively high. In future work, each module will be integrated into a system to realize the miniaturization and integration of the monitoring system. Second, the efficiency of the signal processing algorithm is low, and the algorithm needs to be further optimized to improve the working efficiency of the system. Finally, the work of the system in the actual environment is further studied, focusing on the impact of train vibration on the system, and trying to use the train’s environmental signal as the excitation signal.

## Figures and Tables

**Table 1:** Simulation parameters.

Rail Type	Elastic Modulus (Pa)	Poisson’s Ratio (α)	Density (kg/m^3^)	Grid Length (m)	Excitation Signal Frequency (kHz)
CHN60	2.1 × 10^11^	0.29	7850	8.6 × 10^-4^	350

**Table 2:** Performance evaluation metrics and its expression.

Evaluation Metric	Expression	Parameter Description
RMSE	$\mathrm{RMSE}=\sqrt{\frac{\sum_{i}{\left(y_{i}-\hat{y_{i}}\right)}^{2}}{N}}$	$i\mathrm{is}\;\mathrm{the}\;\mathrm{volume}\;\mathrm{of}\;\mathrm{data}\;\mathrm{in}\;\mathrm{the}\;\mathrm{data}\;\mathrm{set},\;y_{i}$$\;\mathrm{is}\;\mathrm{the}\;\mathrm{predicted}\;\mathrm{value},\;\hat{y_{i}}$ is the ideal value, and *N* is the total amount of data.
$R^{2}$	$R^{2}=1-\frac{\sum_{i}{\left(y_{i}-\bar{y_{i}}\right)}^{2}}{\sum_{i}{\left(y_{i}-\hat{y_{i}}\right)}^{2}}$	$i\;\mathrm{is}\;\mathrm{the}\;\mathrm{volume}\;\mathrm{of}\;\mathrm{data}\;\mathrm{in}\;\mathrm{the}\;\mathrm{data}\;\mathrm{set},\;y_{i}$$\;\mathrm{is}\;\mathrm{the}\;\mathrm{predicted}\;\mathrm{value},\;\mathrm{and}\;\hat{y_{i}}$ is the ideal value.
P__Rr010_	$P\text{\_}{\mathrm{Rr}}_{010}=\frac{N_{\mathrm{Rr}010}}{N_{\mathrm{test}}}$	$N_{\mathrm{Rr}010}$$\;\mathrm{is}\;\mathrm{the}\;\mathrm{total}\;\mathrm{number}\;\mathrm{of}\;\mathrm{individual}\;\mathrm{evaluation}\;\mathrm{values}\;\mathrm{with}\;a\;\mathrm{relative}\;\mathrm{error}\;\mathrm{of}\;\mathrm{less}\;\mathrm{than}\;10\%,\;\mathrm{and}\;N_{\mathrm{test}}$ is the total number of test samples.
P__Rr005_	$P\text{\_}{\mathrm{Rr}}_{010}=\frac{N_{\mathrm{Rr}005}}{N_{\mathrm{test}}}$	$N_{\mathrm{Rr}005}$$\mathrm{is}\;\mathrm{the}\;\mathrm{total}\;\mathrm{number}\;\mathrm{of}\;\mathrm{individual}\;\mathrm{evaluation}\;\mathrm{values}\;\mathrm{with}\;a\;\mathrm{relative}\;\mathrm{error}\;\mathrm{of}\;\mathrm{less}\;\mathrm{than}\;5\%,\;\mathrm{and}\;N_{\mathrm{test}}$ is the total number of test samples.

**Table 3:** Time domain feature parameters and their expressions.

Dimensional Feature Parameters		Dimensionless Feature Parameters	
Feature Parameters	Expressions	Feature Parameters	Expressions
Maximum value	$F_{t1}=\max\left(V\left(i\right)\right)$	Kurtosis	$F_{t8}=\frac{\sum_{n=1}^{N}{\left[V\left(i\right)-F_{t3}\right]}^{3}}{\left(N-1\right)F_{t6}^{3}}$
Minimum value	$F_{t2}=\min\left(V\left(i\right)\right)$	Skewness	$F_{t9}=\frac{{\mathrm{NF}}_{t7}}{\sum_{n=1}^{N}\left|V\left(i\right)\right|}$
Average value	$F_{t3}=\bar{V}=\frac{1}{N}\overset{N}{\underset{i=1}{\sum }}V\left(i\right)$	Waveform factor	$F_{t10}=\frac{\max\left|V\left(i\right)\right|}{F_{t7}}$
Square root amplitude	$F_{t4}={\left(\frac{\sqrt{\sum_{i=1}^{N}\left|V\left(i\right)\right|}}{N}\right)}^{2}$	Peak factor	$F_{t11}=\frac{N_{s}\max\left|V\left(i\right)\right|}{\sum_{i=1}^{N}\left|V\left(i\right)\right|}$
Variance biased	$F_{t5}=\frac{1}{N-1}\sum_{i=1}^{N}{\left(V\left(i\right)-\bar{V}\right)}^{2}$	Impulse factor	$F_{t12}=\frac{\max\left|V\left(i\right)\right|}{F_{t4}}$
Standard deviation	$F_{t6}=\sqrt{\frac{\sum_{i=1}^{N}{\left(V\left(i\right)-\bar{V}\right)}^{2}}{N}}$	Margin factor	$F_{t13}=\frac{\max\left|V\left(i\right)\right|}{F_{t7}}$
Root mean square	$F_{t7}=\sqrt{\frac{\sum_{i=1}^{N}{\left(V\left(i\right)\right)}^{2}}{N}}$	Clearance factor	$F_{t14}=\sqrt{\frac{\sum_{i=1}^{N}{\left(V\left(i\right)\right)}^{2}}{N}}$

Parameter description: i is the number of data groups in the data set, and $V\left(i\right)$ is the corresponding signal amplitude. N is the amount of data; $\;\bar{V}$ is the average of the data.

**Table 4:** Frequency domain feature parameters and their expressions.

Feature Parameters	Expressions	Feature Parameters	Expressions
Centroid frequency	$F_{f1}=\frac{\sum_{i=1}^{N}\bar{f}\left(i\right)f\left(i\right)}{2\pi \sum_{i=1}^{N}f{\left(i\right)}^{2}}$	Root mean square frequency	$F_{f2}=\sqrt{\frac{\sum_{i=1}^{N}f{\left(i\right)}^{2}}{4\pi^{2}f{\left(i\right)}^{2}}}$
Mean frequency	$F_{f3}=\frac{1}{N}\sum_{i=1}^{N}f\left(i\right)$	Root variance frequency	$F_{f4}=\sqrt{\frac{\sum_{i=1}^{N}{\left(-F_{f1}\right)}^{2}f\left(i\right)}{\sum_{i=1}^{N}f\left(i\right)}}$

Parameter description: i is the number of data groups in the data set; $f\left(i\right)$ is the corresponding signal frequency; N is the total amount of data.

**Table 5:** Evaluation metric of the 16 path models before reconstruction.

Path	RMSE	R^2^	P__Rr010_	P__Rr005_	Path	RMSE	R^2^	P__Rr010_	P__Rr005_
**E1-R1**	2.2233	0.7529	37.50%	15.63%	**E3-R1**	1.5075	0.8855	37.50%	18.75%
**E1-R2**	1.4772	0.9015	53.13%	40.63%	**E3-R2**	1.8944	0.8199	34.38%	25.00%
**E1-R3**	1.9551	0.8091	53.13%	28.13%	**E3-R3**	0.4755	0.9890	81.25%	59.38%
**E1-R4**	0.6664	0.9782	87.50%	59.38%	**E3-R4**	1.8411	0.8307	43.75%	31.25%
**E2-R1**	1.4222	0.9077	43.75%	15.63%	**E4-R1**	0.8257	0.9660	78.13%	37.50%
**E2-R2**	1.6733	0.8602	56.25%	21.88%	**E4-R2**	1.8289	0.8380	43.75%	31.25%
**E2-R3**	0.7039	0.9751	81.25%	62.50%	**E4-R3**	0.4366	0.9910	100%	84.38%
**E2-R4**	0.7460	0.9735	81.38%	68.75%	**E4-R4**	1.1635	0.9326	62.50%	53.13%

**Table 6:** Evaluation metrics of the multi-path combination after reconstruction.

Path	RMSE	R^2^	P__Rr010_	P__Rr005_
**E-R(optimal)**	0.3762	0.9932	100%	87.50%

## Data Availability

Not applicable.
